# Thoracolumbar retrolaminar block in seven dogs undergoing spinal surgery

**DOI:** 10.1186/s13620-022-00224-7

**Published:** 2022-08-13

**Authors:** Kyratsoula Pentsou, Vilhelmiina Huuskonen

**Affiliations:** UCD Veterinary Hospital, School of Veterinary Medicine, University College Dublin, Belfield, Dublin, D04 W6F6 Ireland

**Keywords:** Retrolaminar block, Hemilaminectomy, Thoracolumbar analgesia, Dogs, Intervertebral disc extrusion

## Abstract

**Background:**

Thoracolumbar intervertebral disc extrusion is a common neurologic complaint in dogs and is associated with debilitating pain that requires careful analgesic management to avoid the transition to a chronic pain state. Recently, there has been an increased effort to incorporate regional anaesthetic techniques whenever possible, both for perioperative analgesia management and for prevention of chronic pain. A novel regional anaesthetic technique named retrolaminar block is a fascial plane block where the local anaesthetic is injected directly on top of the dorsal aspect of the vertebral lamina, in the fascial plane between the lamina and the epaxial muscles. The technique was recently described in humans and it is claimed to provide analgesia in patients undergoing thoracic and lumbar procedures. To the authors’ knowledge, the retrolaminar block has not been previously reported in live dogs.

**Case presentation:**

Seven dogs presented to our hospital for suspected thoracolumbar intervertebral disc extrusion were anaesthetised using an anaesthetic premedication and induction protocol tailored for each individual animal. Once the suspected diagnosis was confirmed, all seven dogs were placed in sternal recumbency, and the target thoracolumbar vertebral spinous process was identified with palpation. A unilateral retrolaminar block was performed in all dogs with 2 mg/kg of 0.25% bupivacaine. Physiologic parameters, as well as responses to nociceptive stimuli, were monitored throughout the anaesthetic event.

Intraoperatively, one dog required a bolus of fentanyl to control nociceptive stimulation while the epaxial muscles were retracted. No further intraoperative rescue analgesia was required in any of the cases. The postoperative pain was assessed using the Short Form of Glasgow Composite Measure Pain Scale for dogs every four hours for the duration of the dogs’ hospitalization. The retrolaminar block reduced the intraoperative requirement for systemic opioids and other adjunct analgesic agents and all dogs were comfortable throughout their hospitalization and up until the time of their discharge.

**Conclusions:**

This case report presents the performance of the retrolaminar block technique as part of multimodal analgesia management in seven dogs undergoing thoracolumbar spinal surgery.

**Supplementary Information:**

The online version contains supplementary material available at 10.1186/s13620-022-00224-7.

## Background

Intervertebral disc extrusion (IVDE), otherwise known as Hansen type I intervertebral disc disease [1], refers to the displacement of nuclear contents of a degenerated intervertebral disc into the vertebral canal [2], causing variable spinal cord and spinal nerve compression. It is the most common cause of spinal cord injury [3] and acute paralysis in dogs [4] and is considered one of the most frequent neurological complaints in veterinary medicine [5]. The evolution of modern imaging modalities such as magnetic resonance imaging (MRI) and computed tomography (CT) have improved accuracy in diagnosis, localisation and evaluation of the severity of IVDE [6]. Thoracolumbar IVDE in particular has been reported to affect 68–87% of dogs that present with IVDE [7], and surgical intervention is often warranted [5].

Thoracolumbar IVDE results in acute severe pain states that combine nociceptive, inflammatory, and neuropathic components [8, 9]. Neuropathic pain in IVDE is a result of the spinal cord injury from the extruded disc material and from the surgical manipulation [8, 10]. Neuropathic pain can be difficult to recognise [11] and failure to treat it adequately can lead to chronic pain states and central sensitisation [10]. In humans, chronic pain left untreated can result in persistent postsurgical pain [12], while increased severity of acute postoperative pain immediately after surgery, is also associated with the development of persistent postsurgical pain [13].

Many analgesic therapies have been employed in the past decades in an attempt to manage pain resulting from IVDE in dogs [14–17], but these treatments mainly target the nociceptive pain component [8]. Local anaesthetics are the only drugs that can produce complete blockade of the nociceptive stimulus in the sensory nerve fibres [18], and evidence shows that regional anaesthetic techniques can decrease the incidence of chronic postsurgical pain [19]. Overall, the incorporation of regional anaesthetic techniques to general anaesthetic procedures has been associated with better perioperative outcomes and greater satisfaction in human patients [20, 21].

**Fig. 1:**
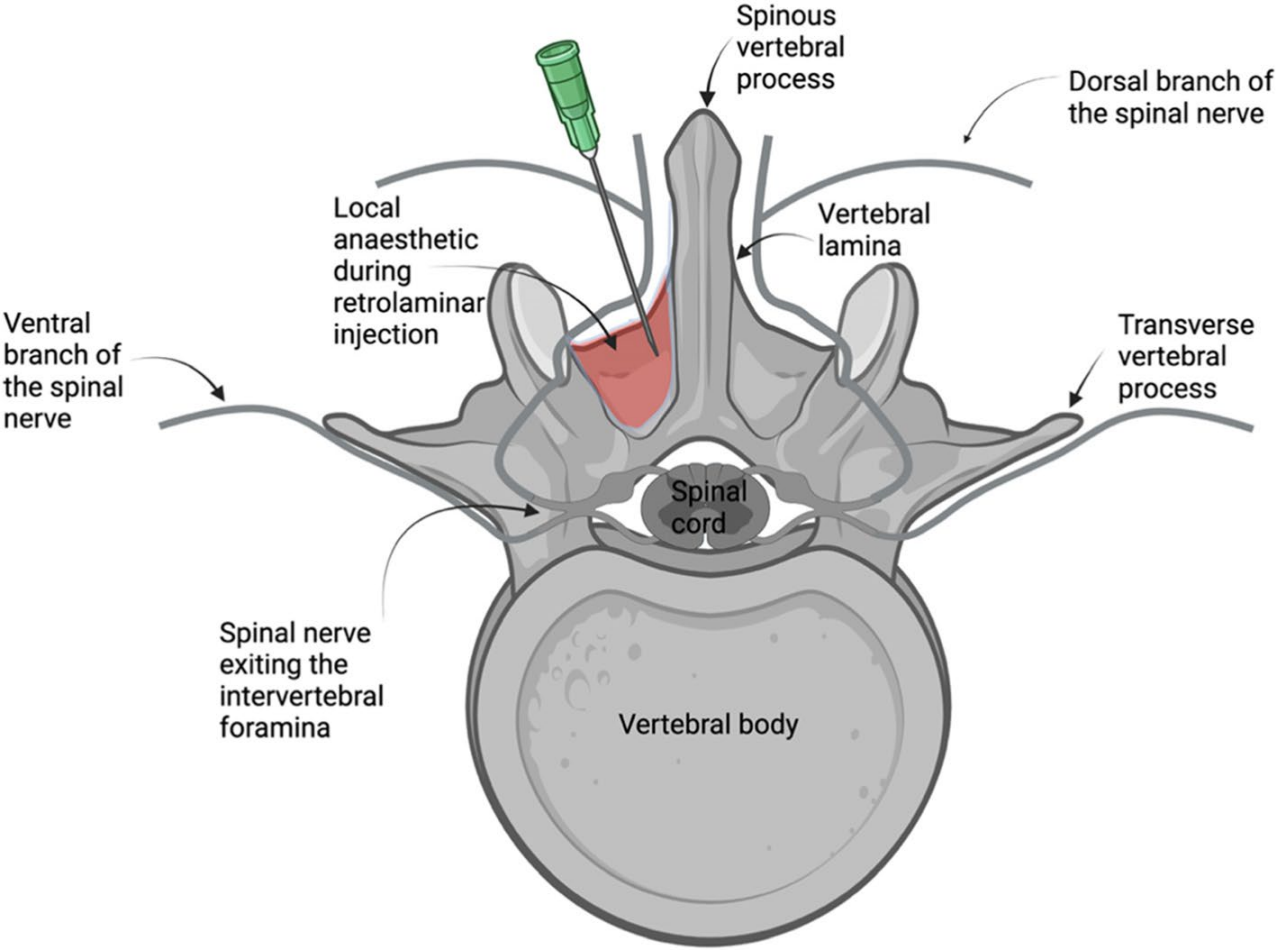
Retrolaminar block: the approach. Schematic representation of retrolaminar injection on a lumbar vertebra. The green needle on the left indicates the position of the tip of the needle as it contacts the dorsal lamina surface. The red coloured area represents the local anaesthetic deposition between the lamina and the epaxial muscles during the retrolaminar injection. (Created by KP with BioRender.com)

Fascial plane blocks are new regional anaesthetic techniques [22], where local anaesthetic is deposited into a tissue plane from which it can spread to adjacent compartments that contain nerves [23]. A new fascial plane block, the retrolaminar block, was developed as a safer and easier alternative to the thoracic paravertebral block in humans [24]. The technique consists of the administration of local anaesthetic directly on the thoracic vertebral lamina, between the lamina and the overlying epaxial muscles [25] (Fig. 1). It is believed that the analgesic action of the block is due to the spread of the local anaesthetic in the paravertebral space where the spinal nerves emerge, and also due to the blockade of the dorsal branch of the spinal nerve as it crosses the epaxial muscles [26], thus providing analgesia for procedures involving the vertebral lamina, the facet joints and epaxial muscles [27]. Clinical studies in humans have presented its analgesic properties in conditions such as mastectomies, rib fractures, and laparoscopies [28–30], while a recent case report in human literature has described successful analgesic management of spinal surgery when the retrolaminar block was included in the protocol [31]. This case report describes the utilisation of the thoracolumbar retrolaminar technique as part of a multimodal analgesia protocol in dogs that underwent spinal surgery for thoracolumbar intervertebral disc extrusion.

## Case presentation

Seven client-owned dogs were referred to University College Dublin Veterinary Hospital for diagnostic investigations of acute ambulatory disturbance, and potential surgery. A thorough physical, orthopaedic, and neurological examination was performed in all dogs. Physical evaluation did not reveal any concurrent disease processes for any of the dogs. Following initial investigations, all animals were transferred to the anaesthesia department to receive a general anaesthetic for further magnetic resonance imaging (MRI) study, with or without contrast, followed by either left-sided or right-sided thoracolumbar mini-hemilaminectomy. Six dogs had both procedures done under the same anaesthetic event, while one dog had surgery on the following day.

Once informed owner consent was obtained, the dogs received either intramuscular (IM) or intravenous (IV) premedication, depending on their demeanour and intravascular catheter presence. After an adequate level of sedation was achieved, an intravenous catheter was placed where needed, and a blood sample for haematology and biochemistry investigation was collected. Preoxygenation with a tight-fitting mask for approximately 5 min with 100% oxygen (O_2_) was performed in all seven dogs and anaesthesia was induced immediately after with propofol^1^ or a combination of propofol and ketamine^2^ administered IV to allow tracheal intubation. Anaesthesia was maintained with sevoflurane^3^ on 100% O_2_ delivered via a circle system. At the same time, all dogs received supportive intravenous fluid therapy with a balanced crystalloid solution (Hartmann’s solution)^4^. All dogs were mechanically ventilated throughout the MRI and the surgical procedure to prevent hypercapnia. Depth of anaesthesia was monitored by checking palpebral reflex, eye position, jaw tone and response to stimuli. Physiologic parameters such as end-tidal carbon dioxide (EtCO_2_)^5,6^, haemoglobin saturation (SpO_2_)^6^ end-tidal sevoflurane concentration (EtSevo)^5,6^, heart rate (HR)^5,6^ and electrocardiogram (ECG)^6^, respiratory rate (RR)^5,6^, oesophageal temperature (T^o^)^6^ and arterial blood pressure^6,7,8^ (either non-invasively, NIBP, using an oscillometric method, or invasively, IBP) were monitored continuously and recorded every five minutes. During the MRI, an MRI-compatible heating mat^9^ was placed over the animals, while during surgical preparation and surgery a forced-air warming blanket^10^ was used to maintain normothermia.

**Fig. 2:**
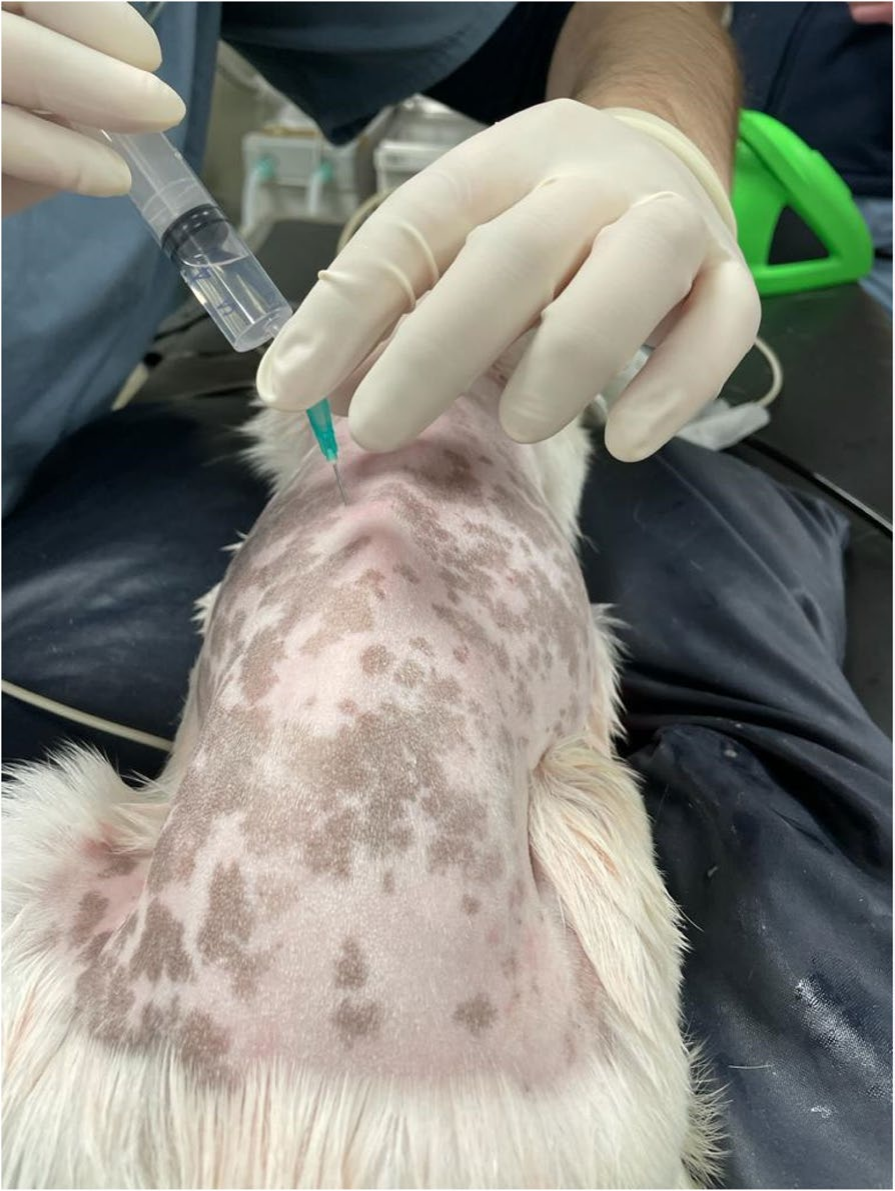
Photographic view of a T13 left-sided retrolaminar injection, performed in Case 1. The puncture site is located approximately 1 cm lateral to the midline at the level of the spinous process of T13. The 21G, 1.4-inch hypodermic needle should be advanced at a 45^O^ angle to the skin, in a caudoventral direction through the epaxial muscles until contact with the lamina is achieved and should remain in a strict parasagittal plane to avoid risk of inadvertent pleural (further lateral from the spinous process) or epidural injection (further medial to the spinous process). After contact with the lamina is achieved, the syringe is attached to the needle and bupivacaine is injected once negative blood aspiration is confirmed

MRI confirmed the diagnosis in all cases, with intervertebral disc extrusion (Hansen type I) in the thoracolumbar area. Prior to the surgical procedure, all dogs were placed in sternal recumbency and once the thoracolumbar area was aseptically prepared, a retrolaminar block was performed on the ipsilateral side of the intended surgery. The wings of the ileum and the spinous process of the sixth lumbar vertebra were identified with palpation, and the lumbar and thoracic spinous processes were palpated cranially. Once the vertebral spinous process of interest was located, a 21G 1.4-inch hypodermic needle^11^ was inserted through the epaxial muscles, in a parasagittal plane, approximately 1 cm lateral to the spinous process of the vertebra where the disc extrusion was identified, in a caudoventral direction and with a 45^o^ angle to the skin, aiming to contact the target vertebra lamina. Once contact of the needle tip with the lamina was achieved, 2 mg/kg of 0.25% bupivacaine^12^ was injected incrementally, while negative aspiration of blood was confirmed (Fig. 2). No adverse effects associated with the injection, such as blood aspiration or resistance to injection, were noted during the performance of the retrolaminar block. All dogs were then moved to theatre and underwent a mini-hemilaminectomy surgery in the thoracolumbar area.

In terms of multimodal analgesia, all seven dogs received methadone^13^, four dogs received medetomidine^14^, and two dogs received ketamine as part of their premedication; six dogs received ketamine as part of their anaesthetic induction; all dogs except for Case 1 received morphine^15^ “splash” in the spinal canal before the closing of the incision; and all dogs received paracetamol^16^ and either meloxicam^17^ or robenacoxib^18^ IV or per os during the perioperative period. No morphine splash was performed on Case 1 as the patient’s nervous demeanour raised concerns for appropriate urinary management in the event of urinary retention [32]. During the surgical procedure, responses associated with nociception were defined as a > 20% increase from baseline of either heart rate, respiratory rate or mean and/or systolic blood pressure and were treated with fentanyl^19^ 2 μg/kg IV bolus. The plan was that if three fentanyl boluses were needed, the dog would be started on fentanyl continuous rate infusion (CRI) ± ketamine CRI. However, only one dog required a rescue fentanyl bolus, as its heart rate increased when the epaxial muscles were retracted. No further rescue analgesia was needed.

Intraoperative complications included bradycardia in six dogs, atrioventricular second-degree block in one dog and hypotension (MAP < 60 mmHg) in four dogs which resolved after treatment with a bolus of glycopyrrolate^20^ 10 μg/kg IV or dobutamine^21^ infusion. All seven dogs were hypothermic during the procedure, but the hypothermia was successfully managed intraoperatively. All dogs recovered smoothly from the anaesthetic event and appeared comfortable throughout the perioperative period. The dogs were monitored closely postoperatively and were moved back to the surgical ward once fully recovered. Postoperative pain was assessed using the short form of Glasgow Composite Measure Pain Scale (GCMPS-SF) for dogs every four hours for the duration of the hospitalization. All dogs were discharged to their owners after 2–11 days depending on their disease progression and ambulatory ability.

Additional information about signalment, the anaesthetic protocol, intraoperative complications, duration of general anaesthesia, heart rate, and mean arterial blood pressure measurements are summarised in Table 1.^1^

**Table 1:** Perioperative anaesthetic and analgesic management, monitoring values and complications in seven dogs undergoing spinal surgery

	Case 1	Case 2	Case 3	Case 4	Case 5	Case 6	Case 7
Signalment	4-year-old Jack Russel terrier mix, male	4-year-old Jack Russel terrier mix, male neutered	9-year-old Jack Russel terrier mix, female spayed	6-year-old Dachshund, male neutered	5-year-old terrier, male neutered	5-year-old Dachshund, female spayed	6-year-old Pekingese, male neutered
Weight (kg)	5.5	7.7	12	12.2	6.5	5.5	12.5
ASA physical status classification	2	2E	2E	2E	2E	2E	2E
Reason for surgery	T13-L1 L-sided IVDE	L1-L2 R-sided IVDE	T11-T12 L-sided IVDE	T11-T12 L-sided IVDE	T12-T13 L-sided IVDE	T12-T13 R-sided IVDE	T13-L1 L-sided IVDE
Body condition score	4/9	7/9	5/9	7/9	7/9	6/9	4/9
Premedication	Methadone (0.3 mg/kg) IV & medetomidine (4 μg/kg) IV	Methadone (0.3 mg/kg) IV & medetomidine (1 μg/kg) IV	Methadone (0.2 mg/kg) IV & medetomidine (1 μg/kg) IV	Methadone (0.3 mg/kg) IM & ketamine (2 mg/kg) IM & acepromazine (3 μg/kg) IM	Methadone (0.3 mg/kg) IM & ketamine (1 mg/kg) IM & acepromazine (3 μg/kg) IM	Methadone (0.3 mg/kg) IV & medetomidine (2 μg/kg) IV	Methadone (0.3 mg/kg) IV & medetomidine (1 μg/kg) IV & maropitant (1 mg/kg) IV
Induction	Propofol (3 mg/kg) IV & ketamine (2 mg/kg) IV	Propofol (2.5 mg/kg) IV & ketamine (2 mg/kg) IV	Propofol (1.5 mg/kg) IV & ketamine (2 mg/kg) IV	Propofol (2.8 mg/kg) IV	Propofol (4 mg/kg) IV & ketamine (2 mg/kg) IV	Propofol (2 mg/kg) IV & ketamine (2 mg/kg) IV	Propofol (2 mg/kg) IV & ketamine (2 mg/kg) IV
Maintenance	Sevoflurane in 100% O_2_	Sevoflurane in 100% O_2_	Sevoflurane in 100% O_2_	Sevoflurane in 100% O_2_	Sevoflurane in 100% O_2_	Sevoflurane in 100% O_2_	Sevoflurane in 100% O_2_
Retrolaminar block (RLB)	T13 (left) RLB with bupivacaine 0.25% (2 mg/kg)	L1 (left) RLB with bupivacaine 0.25% (2 mg/kg)	T11 (left) RLB with bupivacaine 0.25% (2 mg/kg)	T11 (right) RLB with bupivacaine 0.25% (2 mg/kg)	T12 (left) RLB with bupivacaine 0.25% (2 mg/kg)	T12 (right) RLB with bupivacaine 0.25% (2 mg/kg)	T13 (left) RLB with bupivacaine 0.25% (2 mg/kg
Intraoperative rescue analgesia	None	Fentanyl (2 μg/kg) IV once	None	None	None	None	None
0.1 mg/kg morphine “splash” on the spinal canal prior to closure	No	Yes	Yes	Yes	Yes	Yes	Yes
EtSevo (%)	1.6–2.5	1.7–2.2	1.6–2.2	1.9–2.2	1.6–2.3	1.5–2.5	1.3–1.9
HR (bpm)	71–125	38–103	48–90	68–136	55–87	25–87	55–98
Blood pressure monitoring	NIBP	IBP	IBP	IBP	IBP	NIBP	NIBP
MAP (mmHg) measured by NIBP or IBP	62–108 NIBP	67–140 IBP	78–94 IBP	65–122 IBP	73–94 IBP	50–110 NIBP	54–83 NIBP
Intraoperative complications	Hypotension, bradycardia, hypothermia	Hypotension, bradycardia, hypothermia	Bradycardia, hypothermia	2nd degree AV block, hypothermia	Bradycardia, hypothermia	Hypotension, bradycardia, hypothermia	Hypotension, bradycardia, hypothermia
Treatment of intraoperative complications	Glycopyrrolate 10 μg/kg IV	Glycopyrrolate 10 μg/kg IV	None required	Glycopyrrolate 10 μg/kg IV	None required	Glycopyrrolate 10 μg/kg IV & dobutamine CRI 1–2 μg/kg/min	Glycopyrrolate 10 μg/kg IV
Body temperature during the RLB block (°C)	35.5	36.2	36.0	36.2	35.3	36.6	36.1
Postoperative analgesia	Methadone (0.2 mg/kg) IV q4h for 48 h, meloxicam, paracetamol	Methadone (0.2 mg/kg) IV q4h for 48 h, meloxicam, paracetamol	Methadone (0.2 mg/kg) IV q4h for 48 h, meloxicam, paracetamol	Methadone (0.2 mg/kg) IV q4h for 48 h, meloxicam, paracetamol	Methadone (0.2 mg/kg) IV q4h for 48 h, robenacoxib, paracetamol	Methadone (0.2 mg/kg) IV q4h for 48 h, paracetamol	Methadone (0.2 mg/kg) IV q4h for 48 h, meloxicam, paracetamol
Total length of GA (min)	155 (had MRI on the previous day)	251	237	195	180	185	270
Time from induction to the first incision (min)	132	141	128	103	131	130	173
Range of GCMPS-SF scores (first 48 h)	1–6/20	1–4/20	2–4/20	1–2/20	1–2/20	1–2/20	2–4/20
Duration of hospitalisation	5 days	5 days	11 days	8 days	5 days	4 days	2 days

*ASA* American Society of Anesthesiologists, *E* Emergency, *IVDE* Intervertebral disc extrusion, *R* Right, L Left, *T13* Thirteenth thoracic intervertebral space, *L1* First lumbar intervertebral space, *L2* Second lumbar intervertebral space, *T11* Eleventh thoracic intervertebral space, *T12* Twelfth thoracic intervertebral space, *IV* Intravascular, *IM* Intramuscular, *O*_*2*_ Oxygen, *RLB* Retrolaminar block, *EtSevo* End-tidal sevoflurane, *HR* Heart rate, *bpm* Beats per minute, *NIBP* Non-invasive blood pressure, *IBP* Invasive blood pressure, *SAP* Systolic arterial pressure, *MAP* Mean arterial pressure, *DAP* Diastolic arterial pressure, *AV* Atrioventricular, *postop* Postoperatively, *q4h* Every 4 h, *GA* General anaesthesia, *MRI* Magnetic resonance imaging, *GCMPS-SF* Glasgow composite measure pain scale—short form

### Case 1

Case 1 was presented for acute onset of hindlimb ataxia, difficulty on walking and back pain of one-week duration. The patient was anaesthetised for an MRI scan which revealed a left-sided IVDE located between the thirteenth thoracic space (T13) and the first lumbar space (L1). Following the MRI, the dog was recovered from anaesthesia since Case 2 also presented at the same time and was deemed more urgent. During hospitalisation, Case 1 received methadone IV every four hours and a ketamine CRI at 3 μg/kg/min which was stopped 5 h prior to surgery [33]. On the following day, a T13-L1 left-sided mini-hemilaminectomy was performed. Prior to surgery, a left-sided retrolaminar block was performed at the level of T13 with 2 mg/kg of 0.25% bupivacaine. No rescue analgesia was required intraoperatively. Recovery was smooth and uneventful. The highest pain score, 6/20, was measured two hours after the end of anaesthesia and five hours after previous methadone dose and was treated successfully with 0.2 mg/kg methadone IV, after which IV methadone was continued every 4 h for the first 48 h. The dog was discharged with oral paracetamol, meloxicam and gabapentin^22^.

### Case 2

Case 2 was presented for acute onset of hindlimb ataxia. MRI identified IVDE between the first (L1) and second lumbar vertebra (L2) with marked spinal cord compression from the right ventral direction. Surgical intervention was confirmed, and the dog received a right-sided retrolaminar block at the level of L1 with 2 mg/kg of 0.25% bupivacaine. An additional movie file shows the performance of the retrolaminar block in more detail (see Additional file 1). A right-sided L1-L2 mini-hemilaminectomy was performed during which the dog required one dose of fentanyl 2 μg/kg IV for rescue analgesia as an abrupt increase in HR was noted during epaxial muscle retraction. No further intraoperative rescue analgesia was required. The dog’s recovery was smooth and comfortable. Despite consistently low GCMPS-SF scores, the first 0.2 mg/kg dose of postoperative methadone was administered IV 2 h after anaesthesia ended (5.5 h after previous methadone dose) and was continued every 4 h thereafter for the first 48 h. The dog was discharged with oral paracetamol, meloxicam and gabapentin.

### Case 3

Case 3 was presented for acute paraplegia, lethargy, and refusal to move for the past twelve hours. Prior to surgery, the dog received a left-sided retrolaminar block at the level of T11 with 2 mg/kg of 0.25% bupivacaine A left-sided T11-T12 mini-hemilaminectomy was performed, and no intraoperative rescue analgesia was required. The dog’s recovery was very smooth and uneventful. Despite consistently low pain scores, 0.2 mg/kg of methadone was administered IV 3 h after the end of anaesthesia (6.5 h after previous methadone dose) and was continued every 4 h thereafter for the first 48 h of hospitalisation. The dog was discharged with oral paracetamol, meloxicam and gabapentin.

### Case 4

Case 4 was presented for acute onset of hindlimb ataxia and ambulatory paraparesis. A right-sided retrolaminar block at the level of T11 with 2 mg/kg of 0.25% bupivacaine was performed prior to the right-sided T11-T12 mini-hemilaminectomy. No rescue analgesia was required intraoperatively. The dog’s recovery was smooth and comfortable. Despite consistently low pain scores, 0.2 mg/kg of methadone was administered IV 2.5 h after the end of anaesthesia (5 h after previous methadone dose) and was continued every 4 h thereafter for the first 48 h postoperatively. The dog was discharged with oral paracetamol and meloxicam.

### Case 5

Case 5 was presented for acute onset of paraplegia. A left-sided retrolaminar block at the level of T12 with 2 mg/kg of 0.25% bupivacaine was performed prior to the left-sided T12-T13 mini-hemilaminectomy. No rescue analgesia was required intraoperatively. The dog’s recovery was smooth, and it appeared comfortable throughout its hospitalisation period. An additional movie file presents a pain assessment of the dog one hour after the end of the anaesthetic (see Additional file 2). Despite consistently low pain scores, 0.2 mg/kg of methadone was administered IV 1 h after the end of anaesthesia (5 h after previous methadone dose) and was continued every 4 h thereafter for the first 48 h postoperatively. The dog was discharged with oral paracetamol and gabapentin.

### Case 6

Case 6 was presented for acute onset of hindlimb ataxia and paresis after it suffered a fall from a sofa. The dog received a right-sided retrolaminar block at the level of T12 with 2 mg/kg of 0.25% bupivacaine. A right-sided T12-T13 mini-hemilaminectomy was performed and no intraoperative rescue analgesia was needed. Recovery was smooth, and the dog remained comfortable for the remainder of the hospitalisation period. Despite consistently low pain scores, 0.2 mg/kg of methadone was administered IV 3 h after the end of anaesthesia (5 h after previous methadone dose) and was continued every 4 h thereafter for the first 48 h postoperatively. The dog was discharged with oral paracetamol, meloxicam and gabapentin.

### Case 7

Case 7 was presented for acute onset of paraparesis and spinal discomfort. A left-sided retrolaminar block at the level of T13 with 2 mg/kg of 0.25% bupivacaine was performed prior to the left-sided T13-L1 mini-hemilaminectomy. No rescue analgesia was required intraoperatively. The dog recovered smoothly. Despite consistently low pain scores, 0.2 mg/kg of methadone was administered IV 2 h after the end of anaesthesia (6 h after previous methadone dose) and was continued every 4 h thereafter until discharge. An additional movie file shows the ambulation of the patient 20 h after the surgery (see Additional file 3). The dog was discharged with oral paracetamol and robenacoxib.

## Discussion

This case report describes the utilisation of a novel regional anaesthetic technique, the retrolaminar block, as part of multimodal analgesia management in seven dogs that underwent thoracolumbar spinal surgery.

The retrolaminar block has been successful in providing intraoperative thoracic [26] and lumbar [31] analgesia in humans since its development in 2006 [34]. Human cadaveric studies have not yet clarified the exact mechanism of analgesic action of the retrolaminar block [35] and since this regional anaesthetic technique is considered relatively new, its performance is not yet standardized. On those grounds, and to minimise potential risks associated with the use of a novel technique, prior to performing the retrolaminar block in live animals, we investigated the injectate spread after thoracolumbar retrolaminar injections of iodinated contrast-dye mixture in canine cadavers (unpublished material). Preliminary results of our investigations are similar to human cadaveric studies, where the injectate is found to spread into the paravertebral space and surrounding intervertebral foramina, with a variable extent that depends on the volume injected [25, 36, 37]. Thus, we decided to include the retrolaminar block in the analgesic protocol of these seven dogs anaesthetised for spinal surgery, while being fully prepared to treat intraoperative nociception should the block fail to provide adequate antinociception, which is our standard procedure with any local block. This inclusion was deemed successful, as it managed to reduce the overall need for intraoperative administration of opioids and other adjunct analgesic agents that are traditionally used in these cases in our institution. The high level of pain associated with IVDE surgery in dogs warrants a careful analgesic management with a multimodal approach [10]. Multimodal analgesia refers to the use of a combination of different analgesic drugs or techniques, with the aim to enhance the effect of each drug or technique, while at the same time reducing the administered dose and therefore the associated side effects [38]. In our institution, this multimodal approach in dogs suffering from IVDE includes systemic opioids administered in combination with ketamine, lidocaine and/or alpha_2_-receptor agonist infusions [14, 15, 39], paracetamol [40] and/or non-steroidal anti-inflammatory drugs (NSAIDs) [3], and gabapentin [8]. We also commonly use other routes of drug administration such as intrathecal or topical epidural morphine [17, 41, 42].

The inclusion of regional anaesthetic techniques presents a significant advantage as they have been proven to provide better analgesia compared to general anaesthesia with systemic opioids [19], they reduce acute and chronic pain following surgery [19], and overall they offer better outcomes in human patients [19], hence their use is advocated in veterinary medicine as well [43]. With that in mind, several researchers have recently explored the addition of regional anaesthetic techniques in the analgesic management of IVDE, such as peri-incisional infiltration of epaxial muscles with bupivacaine preoperatively and postoperatively [44, 45] and more recently the ultrasound-guided erector spinae plane block (ESP) [46, 47]. While the peri-incisional bupivacaine had contradictory results in the two studies that evaluated the technique, the use of the ESP technique managed successfully to reduce opioid consumption and other pharmacologic interventions [48]. The ESP block is also a newly developed fascial plane block, differing from the retrolaminar block on the injection endpoint, which in the case of ESP block is the transverse process of the spinal vertebra rather than the vertebral lamina [23]. In humans undergoing breast surgery, the ESP and the RLB were found to provide equivalent analgesia [35]. The main difference though lies in the fact that compared to the ESP technique, the retrolaminar technique has been developed and performed successfully in humans also without the aid of ultrasound guidance [24, 30, 34]. Because of that, its performance does not necessarily require expensive equipment or advanced anatomical knowledge, and thus after some training it should be readily accessible to practitioners. Spinal surgeries often happen out of hours when time and resources are limited. On top of that, attempts are being made to minimise the anaesthetic time as much as possible, since prolonged anaesthetic times have been associated with negative outcomes in dogs undergoing surgery for thoracolumbar IVDE. As a result, it might be tempting to avoid technically demanding regional anaesthetic techniques. Even though the addition of ultrasound guidance can increase the accuracy and safety of regional anaesthetic techniques [28], in the case of the retrolaminar block, the risks associated with the technique should at least theoretically be minimal, as no major vessels or nerves exist on the pathway of the needle [30]. Inadvertent epidural injection and deposition of the local anaesthetic in the epidural space is possible after the landmark-guided retrolaminar block [28]. At the same time though, in humans the epidural passive spread of the local anaesthetic is an identified mode of analgesic action of the retrolaminar block even when performed under ultrasound guidance [25].

In humans, one of the problems associated with a single retrolaminar injection is the short duration of analgesia it offers which is limited to the duration of action of the local anaesthetic used [49]. For that reason, in humans, in procedures such as breast surgeries, a retrolaminar catheter is often placed through which repeated doses or continuous infusions of local anaesthetics are administered to completely eliminate [50] or substantially minimise the use of further opioids [24]. Since we were utilising a single injection and not a retrolaminar catheter, and considering that the pain caused by IVDE is very complex and could lead to chronic pain syndrome, central sensitisation and development of persistent postsurgical pain if not adequately treated [10, 12, 13], we could not justify withholding perioperative opioid or anti-inflammatory analgesia. In fact, also human patients that underwent breast surgeries after a single successful RLB or ESP block, still received intraoperative remifentanil infusion and perioperative paracetamol [35].

All dogs in this case report received methadone as part of their premedication. Methadone is a commonly used full mu-agonist opioid, with an intermediate duration of action in dogs (2–4 h) [39] with attributed N-methyl-D-aspartate receptor (NMDA) antagonistic effects more likely to treat neuropathic pain [10], and for this reason, it is commonly used as premedication for spinal surgeries in our institution. The administration of methadone and other analgesic drugs such as medetomidine and ketamine prior to surgery to our dogs could theoretically have prevented the intraoperative response to nociceptive stimuli. However, based on our clinical experience, and considering that most dogs had an MRI scan immediately prior to the surgery prolonging the time between induction and the first incision, the premedication and induction drugs alone would not have been sufficient to prevent intraoperative nociceptive stimulation, while maintaining end-tidal sevoflurane at or below 2.5%. Therefore, we believe that the retrolaminar technique contributed towards intraoperative antinociception. The morphine splash block on the dura at the end of the surgical procedure was most likely responsible for the low postoperative scores noted in our dogs [17]. Although our subjective feeling is that the retrolaminar block also contributed to the immediate postoperative analgesia, without conducting a randomised, blinded clinical trial, we cannot speculate any further.

In this case report, one dog (Case 2), required one dose of intraoperative rescue analgesia when the epaxial muscles were retracted. This could be due to a block failure or an incomplete block. Perhaps a larger volume of local anaesthetic could offer better results as higher volumes of injectate have been associated with a more extensive spread in the paravertebral space in a study with pig cadavers [37]. In our case report, we chose to administer the more diluted 0.25% formulation of bupivacaine with a maximum dose of 2 mg/kg [51]. For the same reason, we chose to perform the retrolaminar block unilaterally, to utilise the highest volume possible, although perhaps a bilateral technique could offer better analgesia. It needs to be noted though that the method we used to decide whether intraoperative rescue analgesia was needed has limitations: even though this is the usual approach used in our institution, there is no supporting evidence in the literature for this practice. Furthermore, invasive blood pressure measurement, which is the gold standard method for monitoring arterial blood pressure intraoperatively, was not available in three out of the seven dogs, one of which was the dog that required rescue analgesia.

A commonly used method to assess the adequacy of intraoperative antinociception is the evaluation of the inhalant anaesthetic sparing effect [52]. The minimum alveolar concentration (MAC) of sevoflurane in dogs, i.e., the alveolar concentration of sevoflurane at which 50% of animals do not move in response to surgical stimulus, is reported to be 2.36% [53]. Without MAC-sparing drugs, end-tidal inhalant concentrations approximately 1.2–1.4 times the MAC are required to maintain a surgical plane of anaesthesia in 95% of the population [54]. In our cases, the end-tidal concentrations of sevoflurane during surgery ranged from 1.3 to 2.5% which is lower than the expected surgical MAC of sevoflurane, suggesting a reduction in sevoflurane requirement. All seven dogs received premedication drugs that are capable of reducing the MAC of inhalant anaesthetics in different degrees, and on top of that, all dogs experienced intraoperative hypothermia that is also known to reduce the MAC of inhalant anaesthetics [55]; however, we believe that the reduction in sevoflurane requirement was at least partially due to the enhanced intraoperative antinociception provided by the retrolaminar block.

One of the limitations of this case report is that the anaesthetic protocols were not standardised but rather tailored for each patient, which could have contributed to the decreased levels of perioperative nociception. A well-designed randomised blinded clinical trial could help to elucidate and quantify more accurately the benefits of the retrolaminar block. Another limitation is the fact that the dogs in our case report were of similar anatomical conformation and size, and perhaps different conformation might hinder landmark identification and make the technique more difficult to perform. An additional limitation is that the postoperative pain scores were assessed by different people. Although everyone who performed the evaluation were appropriately trained veterinary surgeons or nurses, one person performing the pain scores could have reduced the variability between individuals. However, the GCMPS-SF is designed in such a way as to minimise inter-observer variability [56]. Furthermore, the epidural morphine and the administration of postoperative methadone regardless of the low pain scores are limitations that prevented accurate evaluation of any potential opioid-sparing effects of the retrolaminar block. The GCMPS-SF maximum pain score for non-ambulatory dogs is 20. The critical threshold that signifies the need for analgesic intervention is a score equal to or higher than 5 out of 20 [56]. In veterinary clinical practice, there are two strategies that utilise pain assessment tools such as the GCMPS-SF to provide appropriate analgesia [57]. One strategy is to administer analgesia only when the animal manifests signs of pain and exhibits a score equal to or higher than the intervention threshold. This strategy succeeds in limiting the use of unnecessary analgesics, but on the other hand, requires the animal to become painful for an analgesic intervention to occur. The second strategy, and the one utilised in this case report, adopts the prescription of appropriate analgesia (as assessed by the veterinarian designing the analgesic plan) and administration of additional rescue analgesia if the pain score is equal to or above the treatment threshold. Obviously, the second strategy presents the risk of over-prescription of analgesic drugs and increased risk of potential side-effects. However, this strategy might prevent chronic pain establishment [57]. This is an important consideration, as it has been shown that 15% of dogs with thoracolumbar disc extrusion who underwent hemilaminectomy developed chronic neuropathic pain within a year after the procedure, despite attempts to provide appropriate analgesia [3]. For that reason, and since we have no evidence about the actual duration of the analgesic effect provided by the retrolaminar block in dogs, we chose to support postoperative analgesia with both epidural morphine and with IV methadone for the first 48 h, irrespective of the pain scores, to prevent chronic neuropathic pain establishment.

## Conclusion

To conclude, this case report describes the performance of the novel retrolaminar block in seven dogs that underwent thoracolumbar surgery for acute IVDE. The findings encourage future studies evaluating the perioperative analgesic efficacy of the retrolaminar technique in dogs.

### Supplementary Information

**Additional file 1.**

**Additional file 2.**

**Additional file 3.**

## Data Availability

The data are available from the corresponding author on reasonable request.

## Appendix

^1^ Propofol: Propofol-Lipuro 1%, B.Braun Medical INC, Ireland

^2^ Ketamine: Ketamidor, Chanelle Pharma, Ireland

^3^ Sevoflurane: SevoFlo, Zoetis, Belgium

^4^ Hartmann’s solution: Aquapharm 11 Hartmann’s solution for infusion, Duggan Veterinary, Ireland

^5^ GE Healthcare patient monitor: B40 Patient monitor, GE, United States

^6^ Datex OHMEDA patient monitor: Aestiva 5-7900, GE Healthcare, United States

^7^ Cardell 9401 Non-invasive Blood pressure and pulse rate monitor, Mindmark, USA

^8^ Blood pressure transducer: Medex single channel set invasive blood pressure transducer, Henleys Medical Supplies, UK

^9^ MRI safe Thermal Mattress, ACE Veterinary supplies

^10^ Warming device: 3M Bair Hugger Warming Units, Canada

^11^ Disposable injection needles, Kruuse, Denmark

^12^ Bupivacaine 0.25%: Marcain, Polyamp SteriPack, Aspen Pharmacare, Ireland

^13^ Methadone: Synthadon, Animalcare, UK

^14^ Medetomidine: Sedastart, AnimalCare Limited, UK

^15^ Morphine: Morphine sulfate, Mercury Pharma, Ireland

^16^ Paracetamol: Paracetamol, Fresenius Kabi, Dublin, Ireland

^17^ Meloxicam: Loxicom, Norbrook Laboratories Limited, UK

^18^ Robenacoxib: Onsior, NOVARTIS Animal Health Ltd, UK

^19^ Fentanyl: Sublimaze, Piramal Critical Care B.V., Netherlands

^20^ Glycopyrrolate: Glycopyrronium Bromide, Mercury Pharma, Ireland

^21^ Dobutamine: Dobutamine, Hospira, UK

^22^ Gabapentin: Gabin, Gabapentin tablets, Aurobindo Pharma Limited, India

## Abbreviations

CRI: Continuous rate infusion
CT: Computed tomography
ECG: Electrocardiogram
ESP: Erector spinae plane
EtCO_2_: End-tidal carbon dioxide concentration
EtSevo: End-tidal sevoflurane concentration
GCMPS-SF: Glasgow Composite Measure Pain Scale—Short Form
HR: Heart rate
IBP: Invasive blood pressure
IM: Intramuscular
IVDE: Intervertebral disc extrusion
L1: First lumbar space
L2: Second lumbar space
MAC: Minimum alveolar concentration
MRI: Magnetic resonance imaging
NIBP: Non-invasive blood pressure
NMDA: N-methyl-D-aspartate
NSAIDs: Non-steroidal anti-inflammatory drugs
O_2_: Oxygen
Per os: By mouth
RR: Respiratory rate
SpO_2_: Oxygen haemoglobin saturation
T^o^: Temperature
T12: Twelfth thoracic space
T13: Thirteenth thoracic space

## Footnotes

1 *Table **1** to be placed at the end of the case presentation and before the discussion section.*
